# Automatic Defect Description of Railway Track Line Image Based on Dense Captioning

**DOI:** 10.3390/s22176419

**Published:** 2022-08-25

**Authors:** Dehua Wei, Xiukun Wei, Limin Jia

**Affiliations:** 1 School of Traffic and Transportation, Beijing Jiaotong University, Beijing 100044, China; 2 State Key Laboratory of Rail Traffic Control and Safety, Beijing Jiaotong University, Beijing 100044, China

**Keywords:** dense captioning, railway track line defects, automatic defect description, RTLCap, faster RTLCap

## Abstract

The state monitoring of the railway track line is one of the important tasks to ensure the safety of the railway transportation system. While the defect recognition result, that is, the inspection report, is the main basis for the maintenance decision. Most previous attempts have proposed intelligent detection methods to achieve rapid and accurate inspection of the safety state of the railway track line. However, there are few investigations on the automatic generation of inspection reports. Fortunately, inspired by the recent advances and successes in dense captioning, such technologies can be investigated and used to generate textual information on the type, position, status, and interrelationship of the key components from the field images. To this end, based on the work of DenseCap, a railway track line image captioning model (RTLCap for short) is proposed, which replaces VGG16 with ResNet-50-FPN as the backbone of the model to extract more powerful image features. In addition, towards the problems of object occlusion and category imbalance in the field images, Soft-NMS and Focal Loss are applied in RTLCap to promote defect description performance. After that, to improve the image processing speed of RTLCap and reduce the complexity of the model, a reconstructed RTLCap model named Faster RTLCap is presented with the help of YOLOv3. In the encoder part, a multi-level regional feature localization, mapping, and fusion module (MFLMF) are proposed to extract regional features, and an SPP (Spatial Pyramid Pooling) layer is employed after MFLMF to reduce model parameters. As for the decoder part, a stacked LSTM is adopted as the language model for better language representation learning. Both quantitative and qualitative experimental results demonstrate the effectiveness of the proposed methods.

## 1. Introduction

In recent years, the rapid development of rail transit and the fast growth of operating mileage have put forward stricter requirements on transportation safety and maintenance. The health status of the railway track line is the basis for guaranteeing the normal operation of trains and plays a critical role in ensuring the effective, safe, and stable operation of the entire rail transit system [1]. A railway track line is mainly composed of tracks, fasteners, backing plates, and so on. Due to the impact of contact friction and vibration between the train wheels and track, coupled with the influence of the on-site operating environment, defects such as rail corrugation, spalling, and broken fasteners may occur on the railway track line. In practice, railway track line defects have a vital effect on the safety of vehicle operation and passenger comfort and may even further lead to major safety accidents. Furthermore, with the occurrence, evolution, and even deterioration of railway track line defects, the maintenance costs and the difficulty of maintenance decision-making are also increased to some extent. Up till now, the condition monitoring of the railway track line is mainly carried out through manual inspection or the use of track inspection cars, and then the inspection report is manually sorted out and generated according to the inspection results. Nevertheless, manual inspection and generation of inspection reports are of poor efficiency, high cost, and low level of automation and intelligence. In addition, the manufacturing cost of the track inspection car is high, and the track line is occupied during the inspection process. Hence, there is an urgent need to develop a comprehensive railway track line inspection platform that uses advanced technologies such as computer vision, deep learning, and natural language processing to improve the safety and stability of rail transit [2]. Not only can it inspect the railway track lines intelligently, but it can also further generate inspection reports automatically. Moreover, this non-contact sensing monitoring method is more timely, economical, and convenient and can achieve better results and reduce labor.

Over recent decades, researchers in this field have mainly made significant progress in the detection technology of rail surface and fastener defects, but there is no further investigation on the issue of the automatic generation of inspection reports. In the entire condition monitoring system of the railway track line, the inspection report is the main basis for maintenance decision-making. For practical applications, it is necessary to investigate the automatic generation of railway track line inspection reports in text format. At present, with the development of computer vision and natural language processing technology, as well as the continually improving computer technology, it is possible to obtain useful information about multiple objects from images automatically. In particular, dense captioning, a technology based on computer vision, is gaining traction in this field [3,4,5,6,7,8]. Dense captioning is a subset of image captioning technology [9] that understands the characteristics of objects, their activities, and relationships and expresses them in natural language. In comparison with other types of image captioning methods [10], this technology focuses attention on image regions containing objects and generates more objective and detailed region-wise captions. Therefore, it is suitable for analyzing railway track line images with multiple key components and clear positional relationships and generating text information about the type, position, status, and mutual relationship of the key components. Whereas, there are still some problems that need to be solved when developing an automatic defect description method of railway track line image based on dense captioning. First of all, it is difficult to use expressions generated by existing methods (e.g., “black office telephone and pen” or “yellow fire hydrant”) to describe the safety status information of the key components from the field images, which requires constructing its own dataset. Secondly, improved regional feature extraction needs to be developed to achieve more accurate regional localization and better defect description performance. Finally, the processing time of each image should be shortened, and the complexity of the processing progress should be reduced so that the improved method can be applied to practical applications.

To solve these problems, the railway track line image (RTL-I) dataset collected from Beijing Metro Line 6 is constructed firstly, and the images and captions in the dataset are labeled manually in terms of the requirements of maintenance decision-making. In this study, four types of information, including object, type, position, and status, are defined to generate inspection information for the safety status of the railway track line. After that, to improve the defect description accuracy and be more suitable for the scenario of railway track lines, an improved DenseCap model named railway track line image captioning model (RTLCap) is proposed. Specifically, in RTLCap, ResNet-50-FPN [11] is used instead of VGG16 [12] as the backbone of the model to extract better image features. In addition, in response to the issues of object occlusion and category imbalance in the field images, Soft-NMS [13] and Focal Loss [14] are applied in RTLCap to promote the performance of defect description. Finally, to reduce the complexity of RTLCap and speed up the image processing procedure, while maintaining a desirable defect description performance, a reconstructed RTLCap model named Faster RTLCap is presented with the help of YOLOv3 [15]. The proposed Faster RTLCap model follows a similar path to the RTLCap model. More precisely, Darknet53 is used as the basic feature extractor in the encoder part, and a multi-level regional feature localization, mapping, and fusion module (MFLMF) is proposed to extract regional features. Besides, followed by MFLMF, an SPP (Spatial Pyramid Pooling) [16] layer is employed to reduce model parameters. For the decoder part, a stacked LSTM named CM-LSTM is adopted as the language model to improve the defect description performance further. Based on the experimental results, the proposed methods can generate the safety status information of the key components in the railway track line image in a text format effectively and automatically, which are better alternatives for practical applications.

To summarize, the core contributions of this paper are threefold:

1. Based on advanced deep learning networks and natural language processing technologies, the problem of automatic defect description of railway track line image is investigated and solved for the first time, and the proposed methods meet the demand for automatic generation of inspection reports of railway track line safety status.
2. A railway track line image captioning model (RTLCap for short) is proposed based on improved DenseCap, which achieves better defect description accuracy than the original DenseCap and is more suitable for the scenario of the railway track line. To our best knowledge, this is the first research that introduces dense captioning technology into the field of railway track line safety status detection to investigate the automatic generation of inspection reports.
3. Motivated by the work of YOLOv3, a reconstructed RTLCap model named Faster RTLCap is presented. The Faster RTLCap reduces the image processing time effectively while maintaining a sound defect description performance. To be more exactly, the image processing time of Faster RTLCap is about 97.7% faster, and the defect description accuracy is improved by 1.12%.

The remainder of this paper is arranged as follows. In Section 2, some relevant work on rail surface and fastener defects detection methods are reviewed. In Section 3, the proposed railway track line image captioning model (RTLCap) is presented in detail. After that, the redesigned RTLCap model, Faster RTLCap, is investigated in Section 4. Finally, some conclusions of this paper are given in Section 5.

## 2. Related Work

**Figure 1:**
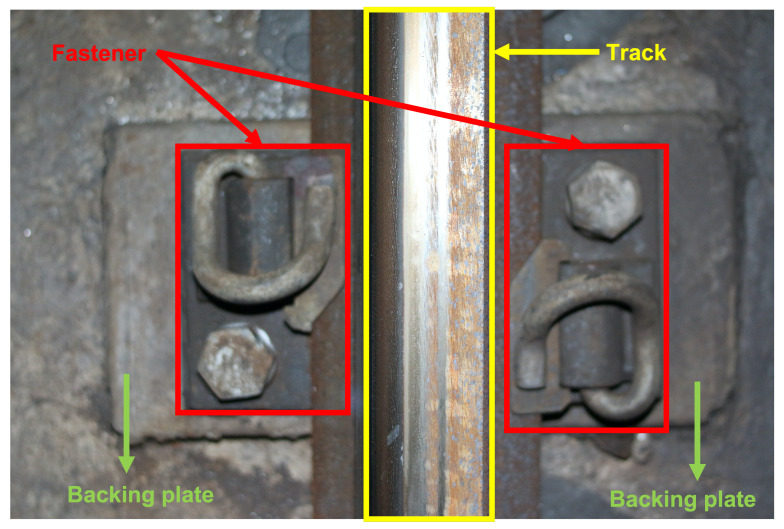
The railway track line structure of Beijing Metro Line 6.

As the lifeline of the whole rail transit system, the safety status of the railway track line directly affects the stability and safety of the operating vehicles during normal driving, as well as the comfort of passengers. Specifically, as shown in Figure 1, the railway track line is mainly composed of key components such as tracks, fasteners, and backing plates. When these key components of the railway track line appear defects and continue to develop and deteriorate, the cost of line maintenance and the difficulty of maintenance decision-making will also increase. To ensure the operational safety and efficiency of rail transit, it is necessary to detect the safety status of key components of railway track lines and generate inspection reports.

In the last decade, a large body of work has been concerned with the development of automated railway inspection methods and systems in terms of advanced technologies. Generally, the detection methods of rail surface and fastener defects mainly contain image processing-based methods and deep learning-based methods. Here, some representative investigations on detection methods based on deep learning are reviewed. The fastener is the key component used to connect the track and backing plate on the railway track line. It ensures that the rail and backing plate are relatively fixed, which is usually in a normal, partially broken, or completely missing state. In [17,18], a multi-layer perception neural classifier is put forward for the detection of missing fasteners and bolts, and an online fastener detection algorithm is implemented with the help of FPGA and GPU. In [19,20], an multitask learning framework (MTL) combined with multiple detectors is proposed to detect railway ties and fasteners, which improves detection performance through intermediate feature sharing and coarse-to-fine detection strategies. In [21], the identification and detection of fastener defects utilizing image processing technologies and deep learning networks are comprehensively investigated, and sound recognition accuracy and recall rate are obtained. In [22], a two-stage framework for detecting defective fasteners is introduced, which is composed of a CenterNet-based fastener positioning module and a VGG-based defect classification module. In [23], a new track fastener detection network architecture called MYOLOv3-Tiny is proposed to enable the deployment of the detection algorithm on lightweight processor devices. In [24], a two-stage classification model based on the modified Faster R-CNN and the support vector data description (SVDD) algorithms is presented for fastener detection, which realizes a fast and accurate detection of four fastener states.

As for the rail surface defect, the main detection methods based on deep learning are as follows. In [25,26], an application of deep convolutional neural networks (DCNNs) for automatic detection of rail surface defects is presented, which achieves non-interference detection. In [27,28], a multiphase deep learning technique is introduced to detect rail surface defects in a vision-based railway track inspection system. Firstly, the track is extracted by image segmentation, and then the extracted track is put into a fine-tuned convolution neural network (CNN) for further classification. In [29], to detect and locate the rail surface defects in real time, two novel rail surface defects detection models with different deep convolutional networks are investigated with the help of MobileNet and YOLOv3. In [30], a track line multi-target defect detection network (TLMDDNet) and DC-TLMDDNet further optimized in the light of DenseNet are proposed, which can detect the defects of track and different types of fasteners simultaneously and comprehensively. In [31], a deep extractor (DE), integrating fully convolutional networks and conditional random fields (CRFs) is put forward for the detection of rail surface discrete defects (RSDDs), which provides new insights in the field. In [32], an attention neural network for rail surface defect detection via CASIoU-guided center-point estimation (CCEANN) is presented to solve the problem of data imbalance and complex situations in actual detection. In [33], an automatic railroad track component inspection method based on instance segmentation is proposed, realizing real-time detection performance in an experimental environment. In [34], an integrated inspection system based on a newly developed rail boundary guidance network (RBGNet) and image processing technologies are constructed, realizing an advanced rail surface segmentation performance. In [35], by using MobileNetv3 and deep separable convolution, an improved YOLOv4 model with lightweight is proposed for railway surface defect detection, enabling real-time detection speed.

It can be concluded that previous studies have shown remarkable progress in the inspection techniques of rail surface and fastener defects, and there is still room for further research in the automatic generation of detection reports.

## 3. Automatic Defect Description of Railway Track Line Image

In this section, the dense captioning model proposed in [3] is reviewed firstly as it forms the foundation for our work. After that, the proposed railway track line image captioning model (RTLCap) is introduced. Finally, the experiments and results are described in details.

### 3.1. Dense Captioning Model

As an extension of image captioning, dense captioning is developed to discover abundant sets of visual contents and to generate captions of wider diversity and more details. The first dense captioning model, named DenseCap, is introduced by the groundbreaking work of Johnson et al. [3], which is the most relevant to our method. DenseCap locates regions of interest and describes them in natural language by performing object detection, soft spatial attention, and image captioning tasks simultaneously in the model.

**Figure 2:**
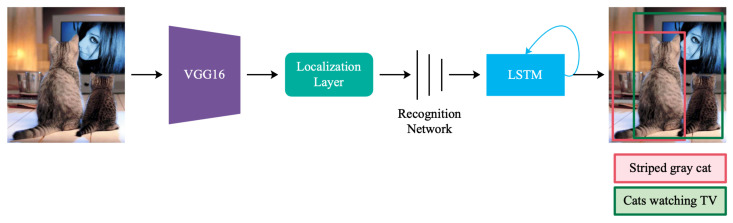
A brief schema of DenseCap.An input image is first processed by the VGG16 [12]. The Localization Layer proposes regions and uses bilinear interpolation to smoothly extract a batch of corresponding activations. After that, these regions are processed using a fully-connected recognition network and described with an LSTM model.

A brief schema of DenseCap is shown in Figure 2. Internally, the input image is first processed by Faster R-CNN [36] with VGG16 [12] as the backbone to obtain object-candidate region features, which are generated by using the soft attention mechanism implemented in the localization layer. Afterward, region features are passed to a fully-connected neural network named as recognition network to get region codes. In the end, the region codes are entered into the Long Short-Term Memory (LSTM) to produce their corresponding sentences. A much more detailed discussion regarding DenseCap can be found in [3].

### 3.2. Railway Track Line Image Captioning Model (RTLCap)

**Figure 3:**
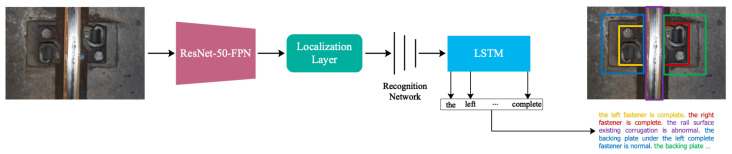
Network architecture of RTLCap. ResNet-50-FPN is used instead of VGG16 as the backbone network.

Inspired by the work of DenseCap, the railway track line image captioning model (RTLCap for short) is proposed in this paper to realize the automatic defect description of the railway track line image. The architecture of RTLCap is shown in Figure 3. To improve the defect description accuracy and be more suitable for the scene of railway track line, RTLCap has made three improvements based on DenseCap, which are discussed in the following.

#### 3.2.1. Backbone and Anchors

As demonstrated in [7,8,37], better feature extraction can greatly improve the performance of description generation tasks. More specifically, accurate feature extraction is conducive to more accurate regional location and better regional description. Therefore, on this basis, ResNet-50, ref. [38] followed by FPN (Feature Pyramid Network), ref. [11] is used as the backbone of RTLCap to extract image features more accurately and comprehensively.

ResNet-50 is a residual learning framework proposed in the light of the existing training deep network, which has the advantages of easy optimization and low computational burden. Moreover, the residual unit is designed to solve the degradation and gradient problems so that the performance of the network can be improved as the depth increases. The Feature Pyramid Network (FPN), proposed by Lin et al. in 2017, was mainly introduced to solve the multi-scale problem in automatic object detection. More precisely, the FPN algorithm promotes the capability of feature expression through the fusion of high-level and low-level feature maps and completes the target detection task on multi-scale feature maps, which improves the detection performance of the model for small targets.

**Figure 4:**
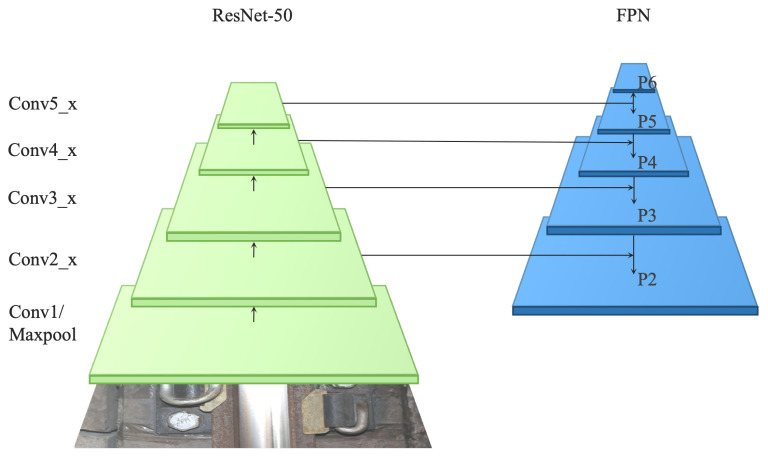
The basic structure of ResNet-50-FPN. (Conv*_x stands for the corresponding Convolutional layer in ResNet-50).

The basic structure of ResNet-50-FPN is shown in Figure 4, which takes a single-scale image of any size as input, and outputs proportionally sized feature maps at multiple levels in a fully convolutional mode. The construction of FPN mainly contains a bottom-up pathway, a top-down pathway, and lateral connections. ResNet-50-FPN has been proved to have a sound performance in [11].

Additionally, anchors are redesigned to be consistent with the scheme used in [11]. In particular, the anchors are defined to have areas of $\left\{32^{2},64^{2},128^{2},256^{2},512^{2}\right\}$ pixels on $\left\{P_{2},P_{3},P_{4},P_{5},P_{6}\right\}$ (as shown in Figure 4), respectively. Furthermore, similar to [36], anchors with multiple aspect ratios {1:2, 1:1, 2:1} are employed at each level. To summarize, there are a total of 15 anchors over the pyramid.

#### 3.2.2. Soft-NMS

**Figure 5:**
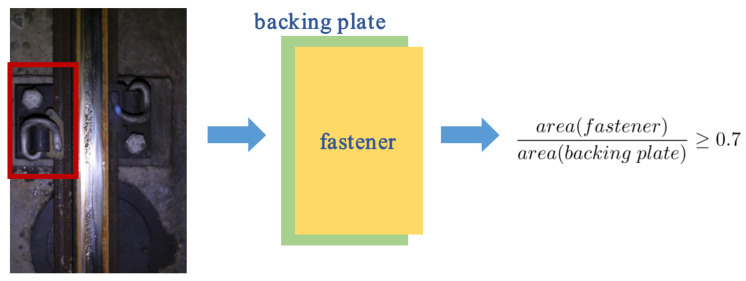
Sketch map of the overlap between the fastener area and the backing plate area.

Non-maximum suppression (NMS) algorithm is an important part of the object detection pipeline. In short, the detection boxes are sorted from high to low according to their scores firstly. Then, the detection box *M* with the maximum score is selected, and all other detection boxes that significantly overlap with *M* are suppressed. At last, this process is applied to the remaining boxes recursively. According to the design of the algorithm, when two target boxes overlap greatly, the box with a lower score is discarded due to the large overlap area with the higher one, resulting in missed detection. In practice, the railway track line images collected from the field face such a problem during the detection process. As shown in Figure 5, in some images, the overlap between the fastener area and the backing plate area is greater than 0.7, which causes the final description result to be incomplete. To solve this problem, the Soft-NMS [13] method is adopted to replace the NMS algorithm used in the original model.

The main idea of Soft-NMS is to reduce the confidence coefficient of the detection boxes that have significant overlap with *M*, instead of discarding them directly. There are two typical rescoring functions for Soft-NMS, linear penalty function, and Gaussian penalty function. Taking into account the continuity of the function, the Gaussian penalty function is employed in our work, which is formulated as follows

(1) $$s_{i}=s_{i}*e^{-\frac{iou{\left(M,b_{i}\right)}^{2}}{\sigma }},\forall b_{i}\notin D$$

where *D* stands for the set of final detections. $b_{i}$ denotes one of the detection boxes and $s_{i}$ is the corresponding confidence coefficient. More details about Soft-NMS can be found in Reference [13].

#### 3.2.3. Focal Loss

In addition to the occlusion problem, the field track line images also meet the challenge of category imbalance. Concretely, in the field railway track line, there are far more key components such as fasteners in the normal state than in the abnormal state, which causes the problem of category imbalance, and affects the precision of the final description results.

**Figure 6:**
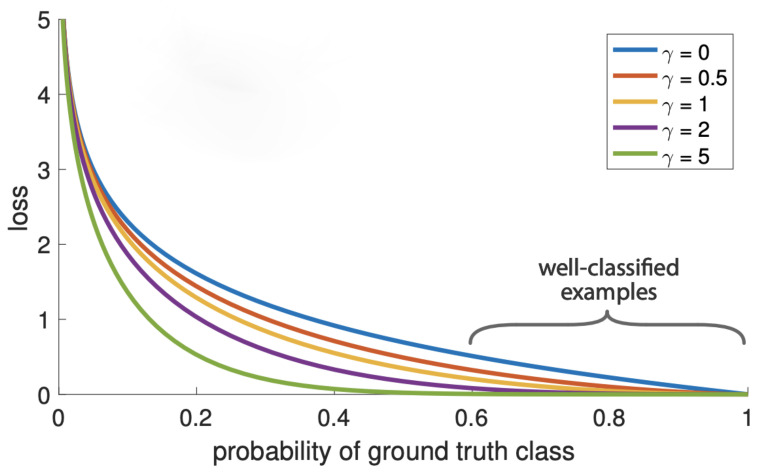
Illustration of the Focal Loss. This figure is from [14].

To address this problem, the Focal Loss (FL) function discussed in [14] is utilized in our work. The FL is modified on the basis of the standard Cross Entropy (CE) loss, which reduces the weight of easy-to-classify samples so that the model can focus more on difficult-to-classify samples during training. More formally, the CE and FL are given by

(2) $$CE\left(p_{t}\right)=-log\left(p_{t}\right)$$

(3) $$FL\left(p_{t}\right)=-{\left(1-p_{t}\right)}^{\gamma }log\left(p_{t}\right)$$

where $p_{t}$ represents the predicted probability of ground truth class. γ≥0 is a tunable focusing parameter. As shown in Figure 6, when setting γ>0, FL reduces the loss of well-classified samples greatly, and pays more attention to misclassified samples.

### 3.3. Experiments and Results

#### 3.3.1. Experimental Environment and Datasets

The proposed methodology is implemented in Torch and PyTorch frameworks on an Ubuntu 18.04 operating system with NVIDIA Titan X [39] GPU, using Lua and Python programming languages. The RTLCap model is trained with an initial learning rate of $1\times e^{-4}$ for the detection task and $1\times e^{-3}$ for the caption task, respectively. Moreover, the adaptive moment estimation (Adam) [40] with exponential decay rates for the first and second moments of 0.9 and 0.999 is adopted to update the weights of the networks. Note that the parameter ‘epsilon’ is set to $5\times e^{-4}$ in this work. Besides, the idea of transfer learning [41] is utilized in that the weights trained through different datasets are used for the weight values initialization.

**Figure 7:**
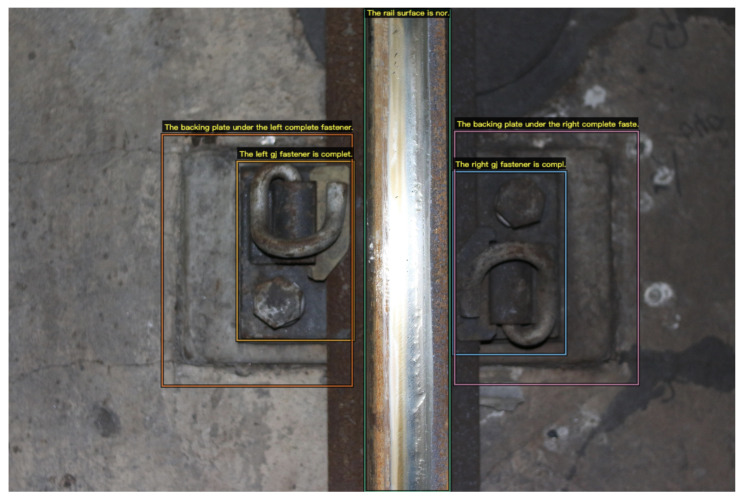
Example of manual labeling using the VGG image annotator with image regions and captions.

In addition, the Visual Genome (VG) [42] dataset and railway track line image (RTL-I) dataset are used as the evaluation benchmarks in our experiments. Similar to [3,43], the images and captions in the datasets are manually annotated using VGG image annotator (VIA) [44] (as shown in Figure 7). Furthermore, by using post-processing operations, the original data exported by VIA is converted into the data format required by the dense captioning model. The details of the Visual Genome (VG) dataset and railway track line image (RTL-I) dataset are as follows.

**VG.** Visual Genome (VG) is the largest dense caption dataset with three available versions now: V1.0, V1.2, and V1.4. Besides, VG has been applied to a variety of vision-language tasks such as dense captioning and Visual Question Answering (VQA) [45]. For the purpose of a fair comparison, the dataset of V1.0 and the same data splits in [3,4] are used. In more details, 77,398 images for training and 5000 images each for validation and test.

**Figure 8:**
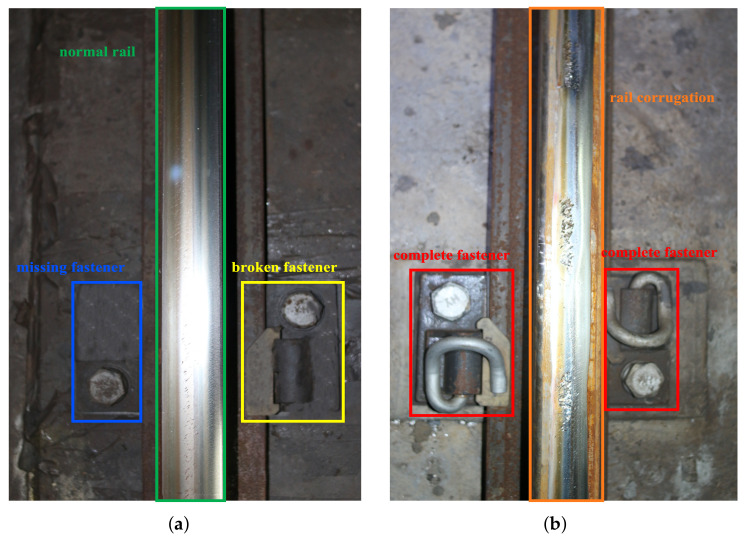
Defects of railway track line of Beijing Metro Line 6. (**a**) missing fastener and broken fastener; (**b**) rail corrugation.

**RTL-I.** Railway track line image (RTL-I) dataset is made up of images that are taken from Beijing Metro Line 6, which is not publicly available in view of its specificity. According to the on-site survey of the Beijing Metro Line 6 and the information provided by the maintenance engineers, the defects of the railway track lines mainly contain three categories: broken fastener, missing fastener, and rail corrugation, as shown in Figure 8a,b, respectively. The rail corrugation is a periodic irregular wear phenomenon on the rail surface. The broken fastener is defined as the complete or partial fracture of the spring bar of the fastener, and the missing fastener is defined as the major or complete absence of fasteners.

In detail, these images are captured by the handhold DSLR camera with the camera angle perpendicular to the roadbed and the distance between the camera and roadbed is kept constant. The collected images are mainly composed of track, fasteners, backing plates and roadbed. Moreover, due to the limited number of key components in defect status, the image data augmentation methods such as rotation, mirroring, noise addition, color perturbation, etc., are probabilistically applied to enhance the RTL-I dataset. Ultimately, RTL-I consists of 1019 images including 4690 captions, each of which corresponds to a region in a given image.

#### 3.3.2. Evaluation Metrics

For evaluation, the prediction results are measured in terms of the mean Average Precision (mAP), which has been used in previous dense captioning works to assess the accuracy of localization and description comprehensively. In more detail, localization accuracy is determined using Intersection over Union (IoU) thresholds, {0.3,0.4,0.5,0.6,0.7}, while description accuracy is determined by METEOR [46] score thresholds, {0,0.05,0.1,0.15,0.2,0.25}. METEOR is employed here not only because it produces the harmonic mean of precision and recall, but because it is considered the most relevant indicator in image description evaluation. The average precision is calculated across all paired settings of the above thresholds and the mAP is reported.

#### 3.3.3. Loss Function

Generally, the loss function is a criterion for evaluating the performance of a model. Following the definition of the loss function in [3], the loss of our framework is also mainly composed of three parts: detection loss ($L_{det}$), bounding box regression loss ($L_{bbox}$), and caption loss ($L_{cap}$). The total loss function of training is as follows

(4) $$L=\alpha L_{det}+\beta L_{bbox}+\gamma L_{cap}$$

(5) $$L_{det}=L_{det\_rpn}+L_{det\_cls}$$

(6) $$L_{bbox}=L_{bbox\_rpn}+L_{bbox\_cls}$$

where $L_{det\_rpn}$ and $L_{det\_cls}$ denote the detection loss in the region proposal network and recognition network, respectively. In the same way, $L_{bbox\_rpn}$ and $L_{bbox\_cls}$ represent the bounding box repression loss in the two networks. More concretely, $L_{det\_rpn}$ is a two-class cross-entropy loss for foreground/background regions, and $L_{det\_cls}$ is the aforementioned focal loss. Both $L_{bbox\_rpn}$ and $L_{bbox\_cls}$ are smoothed-L1 losses. Meanwhile, for the caption loss $L_{cap}$, a cross-entropy loss of sentences for description generation is used. Referring to [3], the weighting coefficients α, β, and γ are set to 1.0 in our experiments.

#### 3.3.4. Availability of the ResNet-50-FPN

In this subsection, the availability of using ResNet-50-FPN as the basic feature extractor in the DenseCap model (denoted as DenseCap_RF) is evaluated on the Visual Genome (VG) dataset. The mAP results are shown in Table 1, in which the larger the mAP value, the better the performance of the model. The DenseCap model introduced in [3] is used as our baseline model which forms the basis of our work. Additionally, the performance of the dense captioning model proposed in [4] is also presented, which incorporates joint inference and visual context fusion (JIVC for short). The best result is highlighted in bold.

As shown in Table 1, in comparison with the most basic two dense captioning models, the ResNet-50-FPN helps to improve mAP scores with gains of 4.38 and 0.46, respectively, indicating the superiority of the DenseCap_RF. On the other hand, it is illustrated again that using a more powerful feature extraction network like ResNet-50-FPN can facilitate the dense captioning task.

#### 3.3.5. Performance Evaluation for RTLCap

In this experiment, by comparing with DenseCap and DenseCap_RF, the validity of our method on the RTL-I dataset is evaluated. The results are shown in Table 2. From these experimental results, it can be concluded that the proposed RTLCap attains the best defect description performance in these models. Specifically, for the RTL-I dataset, the mAP of RTLCap is 0.980, which achieves 0.837 and 0.022 gains compared to DenseCap and DenseCap_RF, respectively. Thus, it is a better alternative for practical applications on railway track lines.

**Figure 9:**
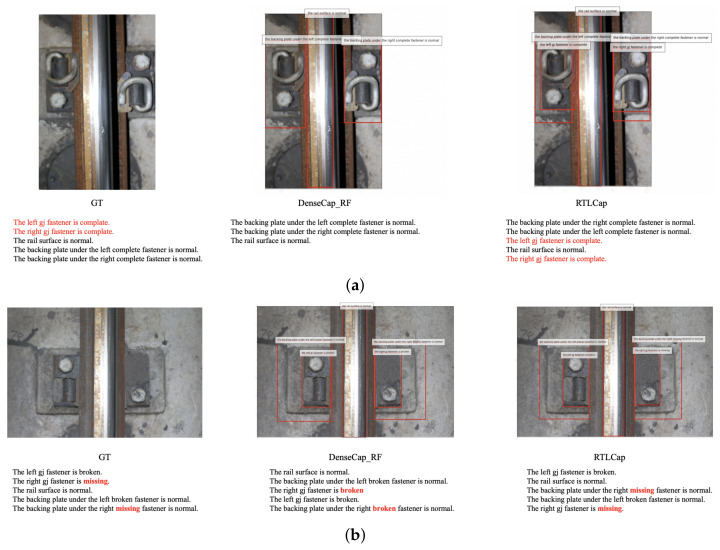
(**a**,**b**) Qualitative comparisons between DenseCap_RF and RTLCap. From left to right are the grounding truth (GT), the prediction of DenseCap_RF and the prediction of RTLCap, respectively. Note that the prediction output is sorted by the confidence score.

Figure 9 shows two examples of comparison results between the DenseCap_RF and the proposed RTLCap, which proves the effectiveness of RTLCap qualitatively in solving the problem of missing descriptions caused by object occlusion and misdescription caused by category imbalance.

## 4. Faster Railway Track Line Image Captioning Model

The RTLCap proposed in Section 3 achieves sound performance and is suitable for the automatic defect description of the railway track line image. Nonetheless, compared to the traditional NMS, the application of Soft-NMS brings a greater time cost to RTLCap, thus slowing down the image processing speed. Moreover, in the light of RPN (Region Proposal Network), the location and encoding of the region features in RTLCap are cumbersome, and there is redundancy in the generation of candidate boxes. Hence, the structure of RTLCap can be further simplified and optimized.

Nowadays, with the continuous in-depth research and development of one-stage object detection algorithms, many tasks completed by the two-stage object detection algorithm can be well fulfilled by the one-stage method. Moreover, recent results show that one-stage methods can achieve a balance of speed and accuracy [47], which presents an opportunity for reconstructing RTLCap to further improve performance.

### 4.1. One-Stage Detection Algorithm

Overall, the object detection algorithm based on deep learning is mainly divided into two streams: two-stage and one-stage algorithms [48]. In detail, the two-stage detection algorithm generates candidate regions on the image firstly, and then performs classification and boundary regression on each candidate region in turn, while the one-stage detection algorithm locates and classifies all targets on the entire image directly, omitting the step of generating candidate regions.

Compared with the two-stage algorithm represented by the R-CNN series [36,49,50,51], the regression-based one-stage algorithm has a simpler detection process, faster reasoning speed, and meets the real-time requirements. More recently, encouraged by recent advances in computer vision and deep learning technologies, many one-stage detection algorithms with sound performance have been proposed, such as SSD series network [52,53,54], YOLO series network [15,55,56,57], RetinaNet [14], etc. Among them, the YOLOv3 proposed by Redmon et al. in 2018 obtained a better speed and accuracy trade-off at that time, and it is also one of the preferred algorithms for object detection in the industry. Therefore, we can borrow recipes from YOLOv3 in redesigning the RTLCap model.

### 4.2. Faster RTLCap

**Figure 10:**
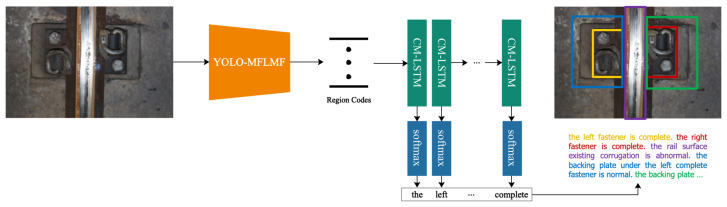
Network architecture of the proposed Faster RTLCap model.

To decrease the complexity of RTLCap and further speed up the processing progress of railway track line images, a reconstructed RTLCap model is introduced in this paper, named Faster RTLCap. More closely, Faster RTLCap follows a similar path to RTLCap, which is mainly composed of two parts, a feature bifurcation-fusion-based encoder part and a stacked LSTM-based decoder part. The architecture of the Faster RTLCap model is shown in Figure 10, and the details of the Faster RTLCap are described below.

#### 4.2.1. Feature Bifurcation-Fusion-Based Encoder Part

**Figure 11:**
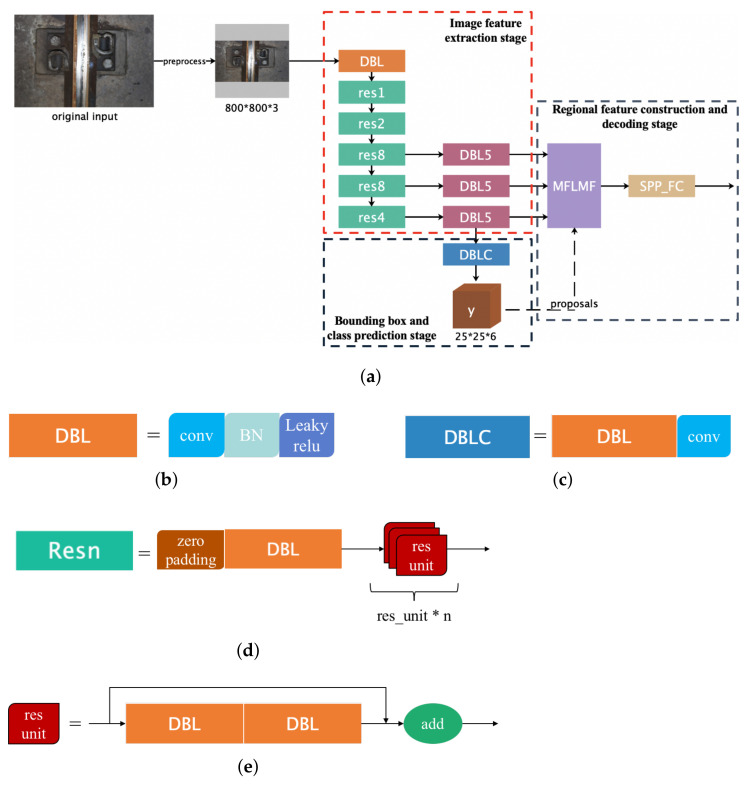
(**a**) The architecture of the bifurcation-fusion-based encoder part (YOLO-MFLMF); (**b**) DBL module; (**c**) DBLC module; (**d**) the module of Resn; (**e**) sketch map of the res unit. Note that the numbers in figure (**a**) indicate the number of blocks or components.

As shown in Figure 11, the encoder part based on bifurcation-fusion (named YOLO-MFLMF) is divided into three steps: image feature extraction stage, bounding box, and class prediction stage, and regional feature construction and encoding stage.

- **Image Feature Extraction Stage**

In the image feature extraction stage, the Darknet53 [15] is used as a feature extractor to obtain basic feature maps of the input image, of which the accuracy is comparable to ResNet-101 and ResNet-152. It consists of 52 convolutional layers and 1 maximum pooling layer. Similar to [15], the final pooling layer of Darknet53 is removed, and the feature maps obtained by the last three Resn module are fed to the corresponding DBL5 modules to further extract and abstract features, respectively. Hence, an input image of shape 3×W×H gives rise to a tensor of features of shape $C^{{}^{\prime }}\times W^{{}^{\prime }}\times H^{{}^{\prime }}$, where $C^{{}^{\prime }}=\left\{128,256,512\right\}$, $W^{{}^{\prime }}=\left\lfloor \frac{W}{k}\right\rfloor$, $H^{{}^{\prime }}=\left\lfloor \frac{H}{k}\right\rfloor$, and k={8,16,32}. Note that $C^{{}^{\prime }}$ and *k* are in one-to-one correspondence. The acquired feature maps are the global features of the image with multi-level receptive fields.

- **Bounding Box and Class Prediction Stage**

**Figure 12:**
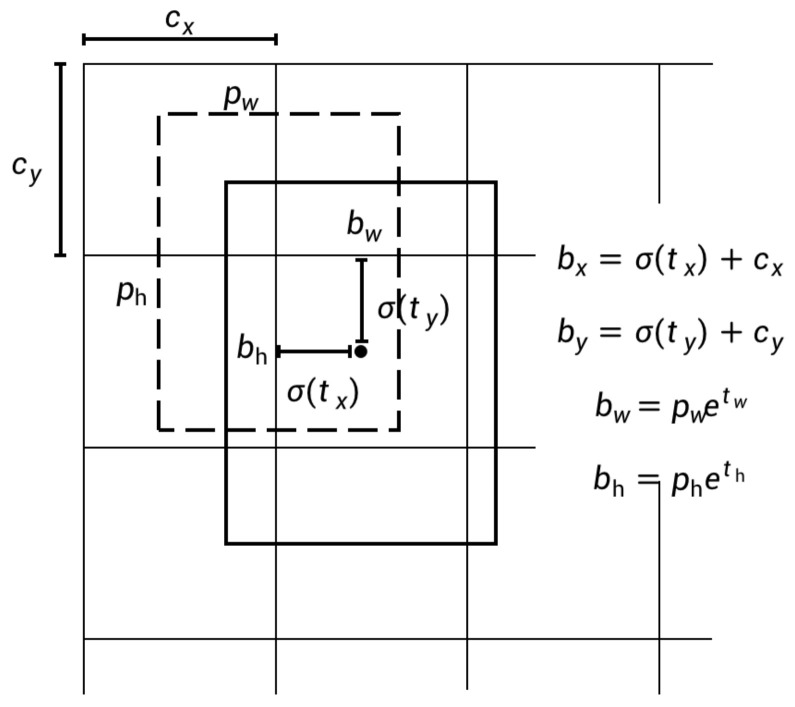
Schematic diagramof calculation of $b_{x}$, $b_{y}$, $b_{w}$, $b_{h}$.

Following [15], the dimensional clusters are applied as anchor boxes to predict bounding boxes. The generation of anchor boxes is outlined in detail later. For bounding box prediction, 4 coordinates are predicted as $\left(t_{x},t_{y},t_{w},t_{h}\right)$ for each bounding box in this stage. Based on this, as shown in Figure 12, the width $b_{w}$, height $b_{h}$ and center coordinates $\left(b_{x},b_{y}\right)$ of the box are calculated as follows

(7) $$b_{x}=\sigma \left(t_{x}\right)+c_{x}$$

(8) $$b_{y}=\sigma \left(t_{y}\right)+c_{y}$$

(9) $$b_{w}=\left(p_{w}\right)e^{t_{w}}$$

(10) $$b_{h}=\left(p_{h}\right)e^{t_{h}}$$

where $c_{x}$ and $c_{y}$ are the offsets of the grid where the center of the target object is located from the first grid coordinate of the detection map. $p_{w}$ and $p_{h}$ represent the width and height of the preset anchor box, respectively. As a result, the predicted positive region proposals with 4 coordinates are input to the next stage as the foundation for regional feature positioning. In addition, as in [15], each box uses multi-label classification in this stage to predict the classes that the bounding box may contain.

- **Regional Feature Construction and Encoding Stage**

**Figure 13:**
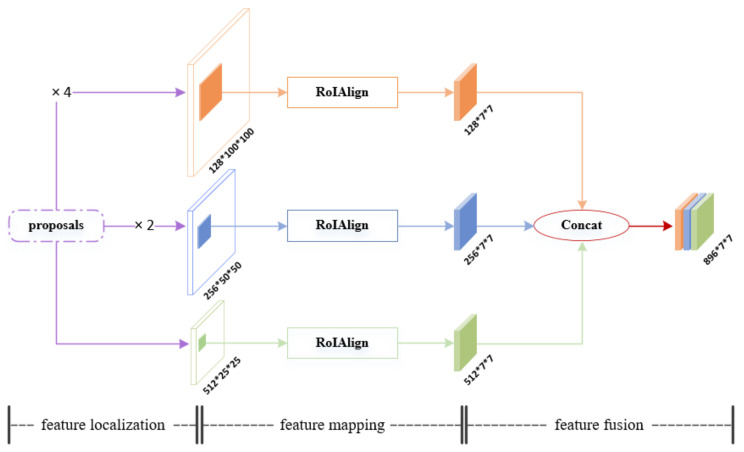
The internal structure of MFLMF.

First of all, in this stage, the basic image feature maps obtained from the image feature extraction stage, and the proposals predicted in the bounding box and class prediction stage, are fed to the MFLMF module together to build regional features. The specific structure of MFLMF is shown in Figure 13. It can be seen that the MFLMF module includes three main parts: feature localization, feature mapping, and feature fusion. The MFLMF module plays a key role in the Faster RTLCap model, which is discussed in detail in Appendix A.

Secondly, the SPP_FC module encodes regional features from the MFLMF module. In detail, the features from each region are processed and flattened into a vector by an SPP (Spatial Pyramid Pooling) layer with three scale pooling windows [16] and then passed through two fully connected layers. In this way, the increase in model parameters caused by MFLMF is reduced, and a code of dimension D=4096 is generated for each region that its visual appearance is comprehensively and compactly encoded.

In the end, the codes for all predicted regions are expressed by a matrix of shape B×D and passed to the language model.

- **Convolutional Anchors**

In terms of the idea in [30], the prior anchor boxes are reconstructed by using K-means clustering, in which the Intersection over Union (IOU) of the rectangular box (represented by $R_{IOU}$) is adopted as the similarity, and the distance function of the cluster is given by

(11) $$d\left(B,C\right)=1-R_{IOU}\left(B,C\right)$$

where *B* and *C* denote the size and center of the rectangular box, respectively. $R_{IOU}\left(B,C\right)$ stands for the IOU between two rectangular boxes.

**Figure 14:**
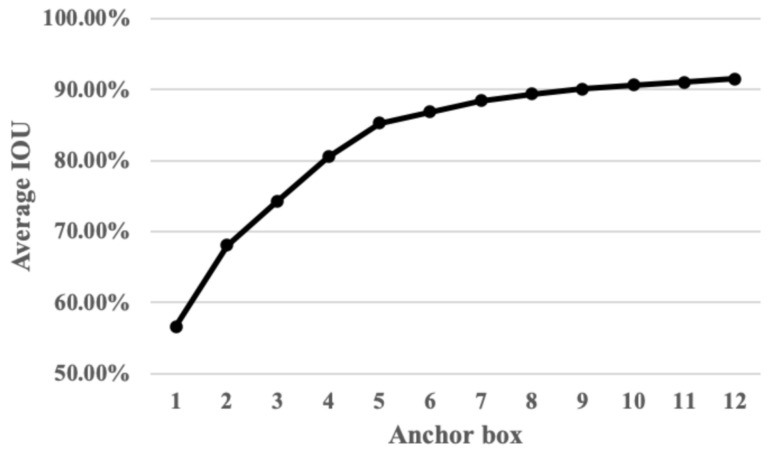
The relationship between the number of anchor boxes and average IOU.

The relationship between the average IOU and the number of anchor boxes is shown in Figure 14. Considering the performance and efficiency of network training, and allowing the prior anchor boxes to better fit the actual size of the target region in the dataset, 9 clusters are chosen as the bounding box priors, which are (104,791), (138,351), (148,792), (163,423), (179,223), (218,239), (230,488), (268,568), and (286,328).

#### 4.2.2. Stacked LSTM-Based Decoder Part

**Figure 15:**
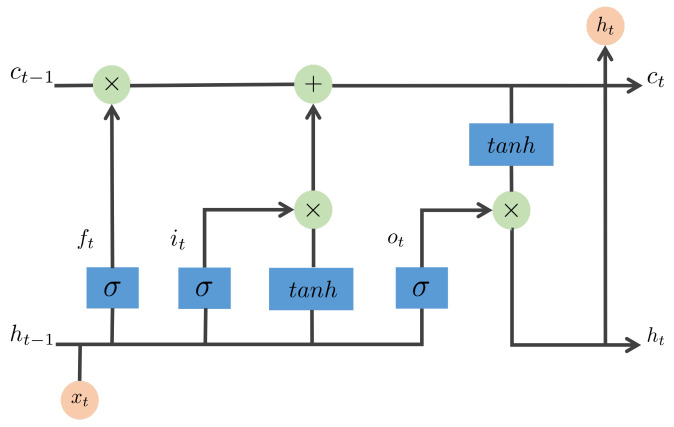
Long Short-Term Memory (LSTM) unit.

For the decoder part, a sentence generator in terms of Long Short-Term Memory (LSTM) cell is considered because it has shown sound performance on sequential tasks such as machine translation and sequence generation [58]. LSTM is a recurrent neural network, which incorporates a built-in memory cell to store information and use long-range context. In detail, as shown in Figure 15, LSTM memory cells are surrounded by three gating units, which are used to control whether to forget the current cell value (forget gate *f*), whether to read its input (input gate *i*), and whether to output the new cell value (output gate *o*), respectively. The definition of the gates and cell update and output are as follows

(12) $$i_{t}=\sigma \left(W_{ix}x_{t}+W_{ih}h_{t-1}\right)$$

(13) $$f_{t}=\sigma \left(W_{fx}x_{t}+W_{fh}h_{t-1}\right)$$

(14) $$o_{t}=\sigma \left(W_{ox}x_{t}+W_{oh}h_{t-1}\right)$$

(15) $$c_{t}=f_{t}⊙c_{t-1}+i_{t}⊙tanh\left(W_{cx}x_{t}+W_{ch}h_{t-1}\right)$$

(16) $$h_{t}=o_{t}⊙tanh\left(c_{t}\right)$$

(17) $$p_{t+1}=Softmax\left(h_{t}\right)$$

where ⊙ denotes the product with a gate value, and the various *W* matrices are the weight parameters to be learned. The nonlinearities are sigmoid σ(·) and hyperbolic tangent tanh(·). The last equation $h_{t}$ is used for feeding to the Softmax function, which produces a probability distribution $p_{t}$ of all words. More details about LSTM can be found in [59].

**Figure 16:**
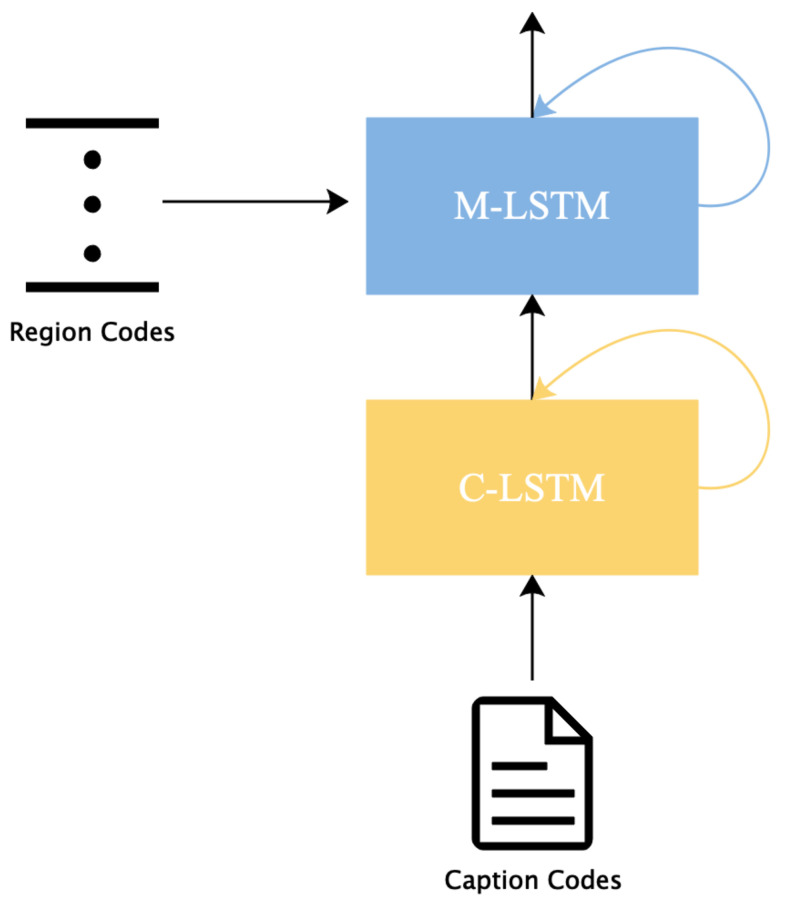
CM-LSTM.

Drawing on the work in [60], a stacked LSTM named CM-LSTM is adopted as the language model in the Faster RTLCap model, as deep architectures have powerful capabilities in feature self-learning [61]. Precisely, as shown in Figure 16, CM-LSTM is comprised of two modules: a Caption-LSTM (C-LSTM) for encoding caption inputs and a Multimodal LSTM (M-LSTM) for embedding visual and textual vectors to a common semantic space and decoding to sentence.

Formally, CM-LSTM works as follows, for raw image input $\tilde{I}$, caption sentence *S*, the encoding performs as

(18) $$I_{t}=\phi \left(\tilde{I};W_{\tilde{I}}\right)$$

(19) $$h_{t\_c}=C\left(ES;W_{c}\right)$$

where ϕ, *C* denote the feature encoding network, C-LSTM model, respectively, and $W_{\tilde{I}}$, $W_{c}$ are their corresponding weights. *E* is embedding matrice learned from the network. Then, the encoded visual and textual representations are embedded to M-LSTM, and the hidden state output of M-LSTM can be formulated as follows

(20) $$h_{t\_m}=M\left(h_{t\_c},I_{t};W_{m}\right)$$

where *M* denotes M-LSTM and its weight $W_{m}$. The visual vector *I* is only fed to the model once, at t=−1. Finally, on the top of the M-LSTM is the Softmax layer, which computes the probability distribution of the next predicted word by

(21) $$p_{t+1}=F\left(h_{t\_m};W_{s},b_{s}\right)$$

where $p\in R^{n}$ and n is the vocabulary size.

### 4.3. Experiments and Results

In this experiment, the proposed model is compared with RTLCap, Faster RTLCap without SPP layer, and Faster RTLCap with LSTM to illustrate the feasibility and effectiveness of Faster RTLCap. More specifically, the RTL-I dataset is used as the evaluation benchmark. The experimental environment and evaluation metrics are the same as discussed in the previous section. Furthermore, the weight values of the feature extraction network in Faster RTLCap are initialized by using the pre-trained YOLOv3 weights trained by MS-COCO [62].

#### 4.3.1. Loss Function

The proposed Faster RTLCap model can be trained in end-to-end by optimizing a joint loss derived from YOLOv3 and RTLCap. Formally, the joint loss *L* is stated as follows

(22) $$L=\alpha L_{coord}+\beta L_{iou}+\gamma L_{class}+\lambda L_{cap}$$

where $L_{coord}$ denotes the coordinate prediction error. $L_{iou}$ represents IoU (Intersection over Union) error. $L_{class}$ stands for the classification error. A specific depiction of these three losses can be found in our preceding work [30]. $L_{cap}$ is a cross-entropy loss, which is the same as the caption loss explained in the previous section. What is more, the values of α, β, γ, and λ are set to 1.0 in our experiments.

#### 4.3.2. Performance Evaluation for Faster RTLCap

The performance comparison results are shown in Table 3, and the best result is highlighted in bold. Faster RTLCap (no SPP) denotes the Faster RTLCap model without the SPP layer, while the Faster RTLCap (with LSTM) represents the decoder part of the Faster RTLCap that uses LSTM. Based on these results, it can be evidently seen that Faster RTLCap has a better defect description performance than RTLCap, despite the small increase in model parameters. More concretely, compared with RTLCap, the image processing time of Faster RTLCap is reduced by about 97.7%, and the defect description accuracy is almost improved by 1.12%. All these results prove the effectiveness of Faster RTLCap, which reduces the image processing time of RTLCap significantly while maintaining an ideal defect description performance. Furthermore, it can also be concluded that the SPP layer used in Faster RTLCap decreases the model parameters effectively and further improves the defect description accuracy. Besides, the experimental results also prove that using CM-LSTM instead of LSTM as the language model improves the defect description performance with a slight increase in model complexity.

#### 4.3.3. The Influence of Choosing Different Numbers of Anchors

To further verify the effect of choosing different numbers of anchors, an experiment is also carried out to evaluate the performance of the Faster RTLCap models with a different number of anchors. The mAP scores and image processing time for Faster RTLCap with different numbers of anchors are shown in Table 4, in which image processing time is obtained by counting and averaging the time of multiple experiments under the same conditions. Based on the experimental results, it can be observed that when the number of anchors is set to 9, Faster RTLCap achieves the highest defect description accuracy. Moreover, the image processing time of Faster RTLCap changes slightly with the number of anchors. Therefore, combined with the analysis of the relationship between the number of anchor boxes and the average IOU (as shown in Figure 14), it can be seen that selecting 9 clusters not only attains a better defect description accuracy, but also achieves an ideal image processing speed.

## 5. Conclusions

In this paper, the issues of automatic defect description of railway track line image are concerned for the first time. First of all, encouraged by recent advances in dense image captioning, the railway track line image captioning model (RTLCap) based on DenseCap [3] is proposed for the considered issues. The experiment on the VG dataset illustrates that using ResNet-50-FPN as the basic feature extractor can promote the dense captioning tasks, while the quantitative and qualitative experiments on the RTL-I dataset demonstrate the performance of RTLCap, and the use of Soft-NMS and Focal loss effectively alleviate the problem of object occlusion and category imbalance. Secondly, to improve the image processing speed and further optimize the structure of RTLCap, a redesigned RTLCap model is constructed usingYOLOv3, named Faster RTLCap. The experimental results indicate that, compared with RTLCap, the method based on Faster RTLCap has better defect description performance, notably reducing the image processing time by about 97.7% and improving the defect description accuracy by 1.12%. Furthermore, the structure of Faster RTLCap is more simplified than that of RTLCap. All findings are in line with our expectations, and the proposed models can automatically generate information about the type, position, status, and interrelationship of key components from the railway track line images collected on the field, providing better alternatives for practical applications.

In future work, we will mainly focus on the following aspects. On the one hand, the RTL-I dataset will be further expanded, and the methods proposed in this work will be further validated in a field test and applied to other different railway scenarios to improve the versatility of these models. On the other hand, the advanced speech recognition and machine translation technologies will be carefully investigated and integrated to develop a comprehensive railway image captioning system, which can not only generate text descriptions of the image content automatically but also generate the corresponding humanized voice descriptions.

## Figures and Tables

**Table 1:** The results on the VG dataset (%).

Method	mAP
DenseCap [3]	5.39
JIVC [4]	9.31
**DenseCap_RF**	**9.77**

**Table 2:** The results on the RTL-I dataset.

Method	mAP
DenseCap [3]	0.143
DenseCap_RF	0.958
**RTLCap**	**0.980**

**Table 3:** The performance comparison results between RTLCap and Faster RTLCap.

Method	mAP	Time (s)	Parameters
RTLCap	0.980	2.0298	**∼94.98 M**
Faster RTLCap (no SPP)	0.984	0.0519	∼211.88 M
Faster RTLCap (with LSTM)	0.986	**0.0363**	∼111.78 M
**Faster RTLCap**	**0.991**	0.0465	∼113.88 M

**Table 4:** The performance of the Faster RTLCap models with different number anchors.

Number of Anchors	mAP	Time (s)
3	0.967	0.0463
5	0.969	0.0469
7	0.963	0.0468
**9**	**0.991**	0.0465

## Data Availability

Not applicable.

## Appendix A. Design Details of MFLMF

As shown in Figure 13, the MFLMF module consists of three parts: feature localization, feature mapping, and feature fusion. Here, the design details of MFLMF are described as follows. Note that for better discussion, the blocks corresponding to the last three Resn modules are named level3, level4, and level5 from top to bottom, respectively.

### Appendix A.1. Feature Localization

Similar to the discussion in [15], the basic feature extractor applied in the Faster RTLCap model is a fully convolutional network. Hence, the relative position of the ROI (region of interest), that is, the target object region, in the feature map at each level is invariant, while its size changes accordingly with the depth of the network.

More formally, let the original input image size and the feature extraction network input size be (img_w,img_h) and (w,h), respectively. In the first step, the original input image is adjusted to the size required by the feature extraction network, with an adjustment factor of $\alpha =min(\frac{w}{img\_w},\frac{h}{img\_h})$. As a result, the size of the adjusted input image (nw,nh) and the offset parameter (dx,dy) are calculated as follows

(A1) nw=⌊img_w∗α⌋

(A2) nh=⌊img_h∗α⌋

(A3) $$dx=\lfloor \frac{w-nw}{2}\rfloor$$

(A4) $$dy=\lfloor \frac{h-nh}{2}\rfloor$$

In terms of these formulas, assuming that the coordinates of a certain point in the original image are (x,y), the position coordinates $\left(x^{{}^{\prime }},y^{{}^{\prime }}\right)$ of this point mapped in a certain level of feature maps can be computed as follows

(A5) $$x^{{}^{\prime }}=\frac{x\times nw}{k\times img\_w}+\frac{dx}{k},k=8,16,32.$$

(A6) $$y^{{}^{\prime }}=\frac{y\times nh}{k\times img\_h}+\frac{dy}{k},k=8,16,32.$$

The reverse is also true; when the coordinates of the predicted proposals are obtained, the regional position coordinates corresponding to the proposals in the feature maps of a certain level in the network can be derived from bottom to top. Therefore, in the light of this inference, after attaining the proposals generated by the bounding box and class prediction stage, localize the feature regions corresponding to the proposals in the feature maps of level3, level4, and level5 as the basis for further constructing regional features (as shown in Figure 13). In this way, the finally obtained regional features can be enhanced by fusing the features with multi-level receptive fields.

### Appendix A.2. Feature Mapping and Fusion

After feature localization, for the sake of realizing the unification of feature sizes, the three-level raw regional features of different sizes are fed to the corresponding RoIAlign layer [51], respectively. To be more exactly, based on the four sampling positions, the RoIAlign layer uses a image bilinear interpolation algorithm to normalize the three-level raw regional features to a certain size, and then pools them to a uniform size.

Finally, the regional features from the three levels are concatenated together along the channel as the final regional features.

## References

[1] Li Y., Trinh H., Haas N., Otto C., Pankanti S. (2013). Rail component detection, optimization, and assessment for automatic rail track inspection. IEEE Trans. Intell. Transp. Syst..

[2] Zuwen L. (2007). Overall comments on track technology of high-speed railway. J. Railw. Eng. Soc..

[3] Johnson J., Karpathy A., Li F.-F. Densecap: Fully convolutional localization networks for dense captioning. Proceedings of the IEEE Conference on Computer Vision and Pattern Recognition.

[4] Yang L., Tang K., Yang J., Li L.J. Dense captioning with joint inference and visual context. Proceedings of the IEEE Conference on Computer Vision and Pattern Recognition.

[5] Wang T.J.J., Tavakoli H.R., Sjöberg M., Laaksonen J. Geometry-aware relational exemplar attention for dense captioning. Proceedings of the 1st International Workshop on Multimodal Understanding and Learning for Embodied Applications.

[6] Yin G., Sheng L., Liu B., Yu N., Wang X., Shao J. Context and attribute grounded dense captioning. Proceedings of the IEEE/CVF Conference on Computer Vision and Pattern Recognition.

[7] Zhang Z., Zhang Y., Shi Y., Yu W., Nie L., He G., Fan Y., Yang Z. (2019). Dense Image Captioning Based on Precise Feature Extraction. International Conference on Neural Information Processing.

[8] Zhao D., Chang Z., Guo S. (2020). Cross-scale fusion detection with global attribute for dense captioning. Neurocomputing.

[9] Vinyals O., Toshev A., Bengio S., Erhan D. Show and tell: A neural image caption generator. Proceedings of the IEEE Conference on Computer Vision and Pattern Recognition.

[10] Hossain M.Z., Sohel F., Shiratuddin M.F., Laga H. (2019). A comprehensive survey of deep learning for image captioning. ACM Comput. Surv. (CsUR).

[11] Lin T.Y., Dollár P., Girshick R., He K., Hariharan B., Belongie S. Feature pyramid networks for object detection. Proceedings of the IEEE Conference on Computer Vision and Pattern Recognition.

[12] Simonyan K., Zisserman A. (2014). Very deep convolutional networks for large-scale image recognition. arXiv.

[13] Bodla N., Singh B., Chellappa R., Davis L.S. Soft-NMS–improving object detection with one line of code. Proceedings of the IEEE International Conference on Computer Vision.

[14] Lin T.Y., Goyal P., Girshick R., He K., Dollár P. Focal loss for dense object detection. Proceedings of the IEEE International Conference on Computer Vision.

[15] Redmon J., Farhadi A. (2018). Yolov3: An incremental improvement. arXiv.

[16] He K., Zhang X., Ren S., Sun J. (2015). Spatial pyramid pooling in deep convolutional networks for visual recognition. IEEE Trans. Pattern Anal. Mach. Intell..

[17] Marino F., Distante A., Mazzeo P.L., Stella E. (2007). A real-time visual inspection system for railway maintenance: Automatic hexagonal-headed bolts detection. IEEE Trans. Syst. Man Cybern. Part C Appl. Rev..

[18] De Ruvo P., Distante A., Stella E., Marino F. A GPU-based vision system for real time detection of fastening elements in railway inspection. Proceedings of the 2009 16th IEEE International Conference on Image Processing (ICIP).

[19] Gibert X., Patel V.M., Chellappa R. Robust fastener detection for autonomous visual railway track inspection. Proceedings of the 2015 IEEE Winter Conference on Applications of Computer Vision.

[20] Gibert X., Patel V.M., Chellappa R. (2016). Deep multitask learning for railway track inspection. IEEE Trans. Intell. Transp. Syst..

[21] Wei X., Yang Z., Liu Y., Wei D., Jia L., Li Y. (2019). Railway track fastener defect detection based on image processing and deep learning techniques: A comparative study. Eng. Appl. Artif. Intell..

[22] Zhou Y., Li X., Chen H. Railway fastener defect detection based on deep convolutional networks. Proceedings of the Eleventh International Conference on Graphics and Image Processing (ICGIP 2019).

[23] Qi H., Xu T., Wang G., Cheng Y., Chen C. (2020). MYOLOv3-Tiny: A new convolutional neural network architecture for real-time detection of track fasteners. Comput. Ind..

[24] Bai T., Yang J., Xu G., Yao D. (2021). An optimized railway fastener detection method based on modified Faster R-CNN. Measurement.

[25] Faghih-Roohi S., Hajizadeh S., Núñez A., Babuska R., De Schutter B. Deep convolutional neural networks for detection of rail surface defects. Proceedings of the 2016 International Joint Conference on Neural Networks (IJCNN).

[26] Liang Z., Zhang H., Liu L., He Z., Zheng K. Defect Detection of Rail Surface with Deep Convolutional Neural Networks. Proceedings of the 2018 13th World Congress on Intelligent Control and Automation (WCICA).

[27] James A., Jie W., Xulei Y., Chenghao Y., Ngan N.B., Yuxin L., Yi S., Chandrasekhar V., Zeng Z. TrackNet-A Deep Learning Based Fault Detection for Railway Track Inspection. Proceedings of the 2018 International Conference on Intelligent Rail Transportation (ICIRT).

[28] Shang L., Yang Q., Wang J., Li S., Lei W. Detection of rail surface defects based on CNN image recognition and classification. Proceedings of the 2018 20th International Conference on Advanced Communication Technology (ICACT).

[29] Feng J.H., Yuan H., Hu Y.Q., Lin J., Liu S.W., Luo X. (2020). Research on deep learning method for rail surface defect detection. IET Electr. Syst. Transp..

[30] Wei X., Wei D., Suo D., Jia L., Li Y. (2020). Multi-target defect identification for railway track line based on image processing and improved YOLOv3 model. IEEE Access.

[31] Zhang Z., Liang M., Wang Z. (2021). A Deep Extractor for Visual Rail Surface Inspection. IEEE Access.

[32] Ni X., Ma Z., Liu J., Shi B., Liu H. (2021). Attention Network for Rail Surface Defect Detection via CASIoU-Guided Center-Point Estimation. IEEE Trans. Ind. Inform..

[33] Guo F., Qian Y., Wu Y., Leng Z., Yu H. (2021). Automatic railroad track components inspection using real-time instance segmentation. Comput.-Aided Civ. Infrastruct. Eng..

[34] Wu Y., Qin Y., Qian Y., Guo F., Wang Z., Jia L. (2022). Hybrid deep learning architecture for rail surface segmentation and surface defect detection. Comput.-Aided Civ. Infrastruct. Eng..

[35] Bai T., Gao J., Yang J., Yao D. (2021). A study on railway surface defects detection based on machine vision. Entropy.

[36] Ren S., He K., Girshick R., Sun J. (2016). Faster R-CNN: Towards real-time object detection with region proposal networks. IEEE Trans. Pattern Anal. Mach. Intell..

[37] Karpathy A., Joulin A., Li F.-F. (2014). Deep fragment embeddings for bidirectional image sentence mapping. arXiv.

[38] He K., Zhang X., Ren S., Sun J. Deep residual learning for image recognition. Proceedings of the IEEE Conference on Computer Vision and Pattern Recognition.

[39] Nickolls J. GPU parallel computing architecture and CUDA programming model. Proceedings of the 2007 IEEE Hot Chips 19 Symposium (HCS).

[40] Kingma D., Ba J. (2014). Adam: A Method for Stochastic Optimization. Comput. Sci..

[41] Geng M., Wang Y., Xiang T., Tian Y. (2016). Deep transfer learning for person re-identification. arXiv.

[42] Krishna R., Zhu Y., Groth O., Johnson J., Hata K., Kravitz J., Chen S., Kalantidis Y., Li L.J., Shamma D.A. (2016). Visual genome: Connecting language and vision using crowdsourced dense image annotations. arXiv.

[43] Bang S., Kim H. (2020). Context-based information generation for managing UAV-acquired data using image captioning. Autom. Constr..

[44] Dutta A., Zisserman A. The VIA annotation software for images, audio and video. Proceedings of the 27th ACM International Conference on Multimedia.

[45] Antol S., Agrawal A., Lu J., Mitchell M., Batra D., Zitnick C.L., Parikh D. Vqa: Visual question answering. Proceedings of the IEEE International Conference on Computer Vision.

[46] Banerjee S., Lavie A. METEOR: An automatic metric for MT evaluation with improved correlation with human judgments. Proceedings of the ACL Workshop on Intrinsic and Extrinsic Evaluation Measures for Machine Translation and/or Summarization.

[47] Liu L., Ouyang W., Wang X., Fieguth P., Chen J., Liu X., Pietikäinen M. (2020). Deep learning for generic object detection: A survey. Int. J. Comput. Vis..

[48] Zou Z., Shi Z., Guo Y., Ye J. (2019). Object detection in 20 years: A survey. arXiv.

[49] Girshick R., Donahue J., Darrell T., Malik J. Rich feature hierarchies for accurate object detection and semantic segmentation. Proceedings of the IEEE Conference on Computer Vision and Pattern Recognition.

[50] Girshick R. Fast r-cnn. Proceedings of the IEEE International Conference on Computer Vision.

[51] He K., Gkioxari G., Dollár P., Girshick R. Mask r-cnn. Proceedings of the IEEE International Conference on Computer Vision.

[52] Liu W., Anguelov D., Erhan D., Szegedy C., Reed S., Fu C.Y., Berg A.C. (2016). Ssd: Single shot multibox detector. European Conference on Computer Vision.

[53] Fu C.Y., Liu W., Ranga A., Tyagi A., Berg A.C. (2017). Dssd: Deconvolutional single shot detector. arXiv.

[54] Li Z., Zhou F. (2017). FSSD: Feature fusion single shot multibox detector. arXiv.

[55] Redmon J., Divvala S., Girshick R., Farhadi A. You only look once: Unified, real-time object detection. Proceedings of the IEEE Conference on Computer Vision and Pattern Recognition.

[56] Redmon J., Farhadi A. YOLO9000: Better, faster, stronger. Proceedings of the IEEE Conference on Computer Vision and Pattern Recognition.

[57] Bochkovskiy A., Wang C.Y., Liao H.Y.M. (2020). Yolov4: Optimal speed and accuracy of object detection. arXiv.

[58] Greff K., Srivastava R.K., Koutník J., Steunebrink B.R., Schmidhuber J. (2016). LSTM: A search space odyssey. IEEE Trans. Neural Netw. Learn. Syst..

[59] Hochreiter S., Schmidhuber J. (1997). Long short-term memory. Neural Comput..

[60] Wang C., Yang H., Bartz C., Meinel C. Image captioning with deep bidirectional LSTMs. Proceedings of the 24th ACM International Conference on Multimedia.

[61] Yu L., Qu J., Gao F., Tian Y. (2019). A novel hierarchical algorithm for bearing fault diagnosis based on stacked LSTM. Shock Vib..

[62] Lin T.Y., Maire M., Belongie S., Hays J., Perona P., Ramanan D., Dollár P., Zitnick C.L. (2014). Microsoft coco: Common objects in context. European Conference on Computer Vision.

